# King Edward's Hospital Fund for London

**Published:** 1905-04-01

**Authors:** 


### KING EDWARD'S HOSPITAL FUND FOR LONDON.

The annual meeting of the General Council was held at Marlborough House on March 29th, the Prince of Wales, President of the Fund, in the Chair.

There were present: The Duke of Norfolk, Lord Rothschild, Sir Joseph Dimsdale, M.P., Sir Trevor Lawrence, Sir John Aird, M.P., Sir William Church (President of the College of Physicians), Mr. C. Stuart Wortley, M.P., Sir Henry Burdett, K.C.B., The Chief Rabbi the Rev. Dr. Adler, Mr. John Tweedy (President of the College of Surgeons), Sir W. J. Collins, Mr. E. A. Cornwall (Chairman of the London County Council), The Rev. Dr. J. Guinness Rogers, The Rev. T. Bowman Stephenson, Mr. Frederick M. Fry, Mr. Albert G. Sandeman, Mr. Edgar Speyer, and the Hon. Secretaries, Sir Savile Crossley, M.P., Sir John Craggs, and Mr. J. Danvers Power.

Lord Rothschild, the Treasurer, in presenting the accounts for the year 1904, said that the total amount received by the Fund had been £99,088; £2,141 was the amount of the expenses of carrying on the work which was equal to only £2 3s. 2d. per cent. on the money received during the year. Of the £99,088, £80,000 was distributed to hospitals and convalescent homes, £13,153 of the available balance represented funds to be held as capital, and £3,793 was carried forward. Though this was a very satisfactory condition of affairs, he should only be too glad if, instead of £99,000, they had £200,000 to deal with. The balance sheet was very satisfactory. On December 31st, 1904, after distributing £80,000, they had investments valued at £664,256 standing in the names of the trustees, and £16,547 at the bankers'. It might be of interest to compare the results of previous years. In 1897 the annual subscriptions were £21,443, in 1904 they amounted to £33,316—an increase of £12,000. They received in 1897 £21,161 on capital account, and virtually nothing in 1904, but in 1902 they received £400,000 for capital. In their first year they distributed £56,826, while last year they were able to distribute £80,000. He desired to express his humble testimony of the marvellous work which the members of the executive committee did in carrying out the wishes of the president.

The Prince of Wales said: My lords and gentlemen,—I beg to move the adoption of the account of receipts and expenditure and the balance-sheet for the year ended December 31st, 1904. This was seconded by the Duke of Norfolk.

Sir Henry Burdett said that the statement of accounts made it very gratifying to remember that when the Fund was first started the idea was that they should have three sources of revenue—subscriptions, capital, and small contributions. They showed that £33,316 had been received in subscriptions. On capital account, with the gifts which had come in this year, and which he hoped would be added to and completed, they expected to be able to count on £50,000 a year as permanent income. The small subscriptions to the League of Mercy had last year resulted in a contribution of £14,000 to the Fund, and he hoped this year they would produce a considerably larger sum. He thought that the figures given by Lord Rothschild showed that the progress of the Fund had been satisfactory, and the progress made had added materially to its strength. That strength was no doubt increased by the circumstance that their expenses last year were less than those of the previous year. (Hear, hear.)

The resolution was carried unanimously.

Sir Savile Crossley read the report of the General Council for the year 1904.

### The Prince of Wales's Speech.

In moving the adoption of the report for the year 1904 it will be advisable first to refer to our financial position. The available income for the year amounted to £85,200, but of this £4,000 is accounted for by the unexpected increase in the sum which we received from the League of Mercy, which was £14,000, against £10,000 in the year 1903. This increase of £4,000 a year is a wonderful testimony to the expansion of the League, and of the growing appreciation of its work by the public. The Council fully realise what a valuable auxiliary it has become to the King's Fund, and I take this opportunity of again expressing my gratitude to all the workers of the League through whose efforts such remarkable results have been achieved. When our grants were considered by the Distribution Committee it was anticipated that our income would only be a little over £80,000, and therefore we fixed that sum for distribution last year. I not only wish to congratulate the League of Mercy on their splendid contribution, but also the Council on our having been able this year to replace part of the sum taken from reserve in the year 1903, when £100,000 was distributed, although the total receipts for that year were under £84,000. Our subscriptions and donations for 1904 show an increase of something under £900, and I sincerely trust that this year the general public will respond even more liberally to our efforts. The amounts due from small sums, though so valuable in the aggregate, have not shown the same increase as the contributions of large sums which we continue to receive. We must attach great importance to adding to the number of our subscribers.

### The Invested Capital Account.

I am, however, especially grateful for the munificence of my ever-generous friend, Lord Mount-Stephen, whose gift of securities to the value of £200,000 was announced after the accounts for 1904 were closed. (Hear, hear.) I also wish to acknowledge with sincere thanks the conditional offer received from Mr. Marshall, which will be equivalent to a gift of £12,000 if his condition is complied with. This condition has reference to our immediate aim, which is to assure for the Fund a permanent income from investments—apart altogether from subscriptions and donations—of £50,000 per annum. With this sum we could practically make sure of being able to distribute £100,000 a year among the London Hospitals. Lord Mount-Stephen's gift brought us to within a capital sum of £30,000 of this goal. Mr. Marshall's offer provides £12,000 of this amount. Sir Charles Tennant has given us £3,000 to capital, so that only £15,000 is required by July 31st to enable us to accomplish our object.

### The King's Original Intention.

But even then we shall not rest on our oars. The King, in his letter written at the inauguration of the Fund, stated that a sum of £100,000 to £150,000 was required annually to place the hospitals on a sound financial basis. Since then the population of London has grown, its building area has been enlarged, hospital requirements have increased. To meet these growing demands I am convinced that our aim must be to provide from the King's Fund an income from all sources which will permit of an annual distribution among the London hospitals of at least £150,000 a year. This was the original intention of the King when he initiated the Fund, and my earnest efforts will be directed towards the fulfilment of that object, which is as near as ever to the heart of the founder. I confidently trust that others will come forward to help us. That their money will be well bestowed they may rest assured, and it is most satisfactory to know that the expenses of the Fund are only a little over two per cent. on the total amount collected, in spite of the increased work we are now undertaking in different directions. (Hear, hear.)

Amalgamation of Small Special Hospitals.

I turn from the question of finance to other matters referred to in the report, the first of which is the object of the amalgamation of small special hospitals. It must not be forgotten that the present number and position of the various hospitals in London are, to a great extent, the chance result of efforts extending over many years, and based on no general scheme under the guidance of one authority. Doubtless this was unavoidable, because no means existed of dealing with the establishment and distribution of London hospitals as a whole, and probably the question had not become pressing when most of the smaller hospitals were founded. The growth of London and the increasing claims of charity have now compelled all who are specially interested in hospital work to consider every suggestion which tends towards either economy or increased usefulness. There is one such suggestion which seems worthy of consideration, namely, whether hospitals which devote themselves to the work of curing one special disease, should not be carried on under as few roofs as possible, and whether, as time goes on, a judicious reduction of the total number of small hospitals might not be advantageously effected. The difficulties of promoting amalgamation, however, are very great. Every one must sympathise with those who, having for many years given their time and money to maintain a hospital in which they are interested, feel reluctant to consent to its absorption by a larger establishment, where its identity would be lost. I need scarcely say that the King's Fund only concerns itself with amalgamations in connection with requests for grants. Our object is not merely to initiate reforms, but rather to act as faithful trustees of the money subscribed by the public through the Fund.

Hospital Economies.

It is not necessary now to add much to the remarks I made on the subject of hospital economies at the distribution meeting last December. All we do is to offer for the consideration of the hospitals any information we are able to provide. It is satisfactory to find that in the great majority of cases our efforts have been received entirely in the spirit in which they were made. I was particularly pleased to observe that the London Hospital sent representatives to inquire into the expenditure and the arrangements of some of the hospitals in Scotland and the North of England, for the purpose of comparison. We shall issue the statistical report again this year, when it will have the benefit of additions and improvements for which we shall be indebted to the co-operation of the hospital managers themselves. This report is as yet only known to the members of the Council and Committees, and to the hospitals directly concerned. I hope that it may be possible some day to make it public; for I believe, by what I hear from those who have studied it, that its usefulness will be generally recognised, and that the credit of its initiation must benefit the King's Fund and attract the sympathies of all who desire that the money they give in charity shall be made to go as far as possible. For the present, however, publication would not be fair to some of the hospitals concerned. A reasonable period must be allowed to enable them to take whatever advantage they can find in the particulars now supplied to them for the first time. But I hope that sooner or later all objections to publication will be removed, and that its usefulness will be extended to hospitals generally

Hospitals and Medical Schools.

Although the report of the Medical Schools Committee, consisting of the Bishop of Stepney and Lord Welby, under the chairmanship of Sir Edward Fry, had not come to hand at the end of last year, I cannot address you to-day without expressing our best thanks to them for having so willingly undertaken the arduous duties which thus devolved upon them. We may indeed congratulate ourselves on our good fortune in securing their valuable services. I also desire to thank with them Mr. Danvers Power, who, at the special request of the Committee acted as their honorary secretary. Considering what weight the report carries, we shall of course endeavour to fulfil its recommendations. The report states that of the 12 hospitals which have medical schools, at least eight contribute from their funds to the support of medical education, and proceeds to declare that the committee "do not find that any special considerations have been advanced in justification of the expenditureby the hospitals on the schools." I am glad, however, to see that the Committee recommends us not to open up past transactions, but to deal only with the future. (Hear, hear.) Inasmuch as the report was not published until after the commencement of the present year, the hospitals cannot act upon it in such a way as to clear the accounts of the present year of these charges. It is hoped, therefore, that we shall be considered to have acted reasonably if we begin with the first complete year after the publication of the report: that is the year 1906. If this is approved, the hospitals will have nine months in which to consider their position. All that we will ask is that the whole of the grants from the King's Fund may go to the relief of the sick poor. If a general account is kept for that exclusive purpose, out of which only hospital payments are made, and into which all ordinary receipts for the general funds of the hospital are paid, then we are satisfied to make our grants to that account. Individual subscribers, and even the whole body of subscribers to a particular hospital, are in a different position from that of subscribers to the King's Fund. It must be remembered that our Fund collects many small sums from people who wish to do something for the sick poor, but who have no special interest in any one hospital, or in anything but the ordinarily accepted objects of the hospitals. While this subject of medical schools remained in doubt, while we were assured that for various reasons it was in the interests of the sick poor that the hospitals should assist in maintaining the schools, we were entitled to accept that view, and we did so. But now that we have in our hands the report of Sir Edward Fry's Committee we are bound to act upon its recommendations, and to consider the policy which we have hitherto followed.

An Institute of Medical Sciences.

I trust and believe that in the near future the result of our action will be of benefit not only to the hospitals themselves but to medical education as well. (Hear, hear.) The Committee point out that while the final studies of a medical student can only be pursued within the walls of a hospital, the teaching of the preliminary and intermediate subjects is more properly carried out in an institution of a university character, and express great satisfaction to find that the statutes of the University of London direct the Senate to "use its best endeavours whenever practicable to secure such common courses of instruction for internal medical students in the preliminary and intermediate portion of their studies under appointed or recognised teachers at one or more centres." I am glad to know that the Senate has already put forward a scheme for the establishment of an Institute of Medical Sciences in connection with the University of London. It is to be hoped that the public will generously support the appeal which has been made for the funds necessary to build and equip such an institute, and thus assist the University in its efforts to provide in London a medical education unrivalled throughout the

Empire. I have observed with satisfaction that since the issue of the Report of the Committee, Mr. Alfred Beit has made a very generous addition to the large donation which he had previously promised to the Institute Appeal Fund.

A Very Important Asset.

Before concluding this review of the year's work I desire to record my feelings of deep gratitude to all those who have assisted in its accomplishment. Remembering that the members of our General Council, the Visiting, Distribution, and Executive Committees, our Honorary Secretaries and other honorary officials are busily occupied either in duties of the State or in their own respective professions, we recognise that their valuable work to the Fund is one of its very important assets. The minute books show that there has been a large and regular attendance at the committee meetings. I know that this entails no small sacrifice of time on the part of the members, and in expressing my appreciation of their services I trust that they will all be willing to continue during the coming year in their various offices. (Hear, hear.)

Other Speakers.

Mr. John Tweedy, President of the Royal College of Surgeons, agreed with His Royal Highness that if this separation of the earlier medical studies from the hospitals were effected, not only would it benefit the hospitals as medical charities, but he was confident it would benefit medical education as well. The two parts of medical studies are quite separate. The preliminary science studies, and studies such as anatomy and physiology, were entirely distinct from the clinical studies. He hoped that, if not within the next year, certainly as soon as practicable this important separation would be carried out, and then in London they would have medical education placed upon the footing which was so much to be desired, and the absence of which those most interested in medical education had had occasion to regret for so many years past.

Mr. E. A. Cornwall, Chairman of the London County Council, said that he had for a long time been aware of and watched with great interest the enormous advantages conferred upon the poor citizens of London by the beneficence of King Edward's Hospital Fund. He had now been greatly pleased to see the great personal interest his Royal Highness took in the Fund, and he could well appreciate the enormous gain it was to London and the London hospitals that they should have that great advantage. Those who were connected with municipal work realised and quite appreciated what his Royal Highness has stated with regard to the Fund and the hospitals—namely, that he would recognise the growing needs of the increasing population. Personally, in addition to being the Chairman of the London County Council, he had for many years represented an East End constituency, and in that capacity he had come into close contact with the very poor. If members of that Council, and no doubt there were some, had had the individual experience he had had for many years with the poor of the East End of London, they would fully realise the great advantage of this Fund to the metropolis. (Hear, hear.)

Indebtedness of Londoners to the King and Prince.

The Rev. T. Bowman Stephenson, D.D., in moving a vote of thanks to the Prince of Wales for presiding, stated that he thought his experience was not shared by any one else in the room, inasmuch as he had lived for 30 years in Bethnal Green. Close acquaintance with the poor by day and night enabled him to look at the work done by the hospitals at an angle of vision which did not readily present itself to others. In his opinion, if London were suddenly deprived of its hospitals a sad and terrible social upheaval would take place. The hospitals in thousands of cases only stood between the despair, not to say desperation, of many who would otherwise have to watch in helplessness the sufferings of those whom they loved. Such people, in consequence of the assistance rendered by the hospitals, were led to see that somewhere there were others who cared for them and were willing to assist them in their troubles. He felt that the Council were enormously indebted to his Royal Highness, and to his illustrious father who founded the Fund, because, if he might say so, his Royal Highness was the one man in England who could do this work, and he thanked God that it was in his Royal Highness's heart to undertake it. (Hear, hear.)

Sir William Collins, in seconding the vote of thanks, said that he agreed with Dr. Stephenson in saying that it would be absolutely impossible to overestimate the value of the interest and support of his Royal Highness in the working of the Fund. He felt sure that his weighty words that day would be duly considered by the authorities of the hospitals. He wished to take the opportunity of thanking his Royal Highness for his close and personal interest in the work of the League of Mercy. He might say that the success of the League was largely due to the great interest, and he might say hospitality, of his Royal Highness the Prince and her Royal Highness the Princess of Wales. (Hear, hear.) He also wished to pay a tribute to the indomitable energy of the presidents, vice-presidents, and members of that League, who had by their personal efforts helped it to its present success. The League constituted the widest possible platform on which everyone could meet and continue the good work of alleviating the sickness and suffering of London. (Hear, hear.)

The vote having been carried by acclamation,

The Prince of Wales said: I thank you, my Lords and Gentlemen, for the very generous reception given to the vote of thanks so kindly proposed and seconded by Dr. Bowman Stephenson and Sir W. Collins respectively. I can only say that as each year the work of the Fund develops, and its influence widens, my interest in it grows keener. I am again authorised to express the King's entire satisfaction at the results achieved during the past year, which is a great encouragement to all of us to continue our efforts to carry out the work which was begun by his Majesty.

